# Variation in the diagnosis and control of hypertension is not explained by conventional variables: Cross-sectional database study in English general practice

**DOI:** 10.1371/journal.pone.0210657

**Published:** 2019-01-10

**Authors:** Rachel Coyle, Michael Feher, Simon Jones, Mark Hamilton, Simon de Lusignan

**Affiliations:** 1 Department of Clinical & Experimental Medicine, University of Surrey, London, United Kingdom; 2 Department of Population Health, Division of Healthcare Delivery Science, NYU School of Medicine, New York, United States of America; 3 Director, Surrey Heartlands Clinical Academy, Guildford, United Kingdom; International University of Health and Welfare, School of Medicine, JAPAN

## Abstract

**Background:**

Hypertension is a major cause of preventable disability and death globally and affects more than one in four adults in England. Unwarranted variation is variation in access, quality, outcome or value which is unexplained by differences in the condition or patient characteristics and which reduces quality and efficiency. Distinguishing unwarranted from variation due to clinical, organisational or patient factors can be challenging. We carried out this study to explore inter-practice variation in the diagnosis and management of hypertension in the Royal College of General Practitioners (RCGP) Research and Surveillance Centre (RSC) network database, a large, representative surveillance database.

**Methods and finding:**

We carried out a cross-sectional study using primary care data extracted from the electronic health records of 1,271,419 adults registered at RCGP RSC general practices on 31^st^ December 2016. Logistic regression was used to indirectly standardise practice-level hypertension prevalence and control against the RCGP RSC population, adjusted for age, gender, ethnicity, deprivation, co-morbidity, NHS region and practice size. Inter-practice variation was demonstrated using funnel plots with 95% and 99.8% control limits. The prevalence of detected hypertension was 18.4% (95% CI 18.4–18.5), n = 234,165. Uncontrolled hypertension was present in 146,553 of 196,052 individuals, 25.2% (25.1–25.4), in whom blood pressure had been recorded in the previous year. Hypertension management varied markedly between practices with a three-fold difference in prevalence, 13.5–38.4%, and a four-fold difference in the proportion of uncontrolled hypertension, 11.8–47.9%. Despite adjustment for sociodemographic and practice characteristics funnel plots demonstrated marked over-dispersion.

**Conclusions:**

Substantial variation in the prevalence of diagnosed hypertension and the management of hypertension was only partially explained by characteristics captured within a routine dataset. The over-dispersion suggests variation is not fully explained by these factors and that context, behaviour and processes of care delivery may contribute to variation. Routine data sources in isolation to not provide sufficient contextual data to diagnose the causes of variation.

## Introduction

Hypertension is internationally a major cause of preventable disability and death globally and England is no exception [1] [2]. In England, hypertension affects more than one in four adults and is the second biggest risk factor for premature morbidity and mortality [3]. However, despite recognition of the importance of hypertension as a risk factor for premature mortality, diagnosis and control of hypertension is suboptimal [4] [5]. Factors associated with uncontrolled hypertension include male gender [6], increased age [6] [7] [8] [9], non-white ethnicity [9] [10] and obesity [6] [9] [11] [12]. Physician and care delivery factors were associated with blood pressure control in cross-sectional studies carried out in the United States [10] [13], however given differences between the UK and US healthcare systems these studies may not be comparable. Evidence from both the UK and internationally demonstrates substantial improvements in the proportion of individuals with diagnosed hypertension who are treated [14] [15] and in the UK there is evidence of improving control of diagnosed hypertension over the last decade [14]. However, under-diagnosis remains problematic and there is scope to reduce hypertension associated cardiovascular disease through improving detection of hypertension [14].

Unwarranted variation in care is variation in access, quality, outcome or value which is unexplained by differences in the condition, or in patient characteristics or preference [16] [17]. Unwarranted variation reduces quality and efficiency through the overuse of inappropriate or ‘low-value’ interventions, and to the underuse of effective ones [16] [18]. The reduction of unwarranted variation in care has long been a focus of quality improvement: monitoring childhood tonsillectomy rates started in the UK in the 1930s [19] and variation continues to be documented in the NHS Atlas of Variation [20] and in the Dartmouth Atlas of Health Care in the USA [21]. However, distinguishing unwarranted from variation due to clinical, organisational or patient factors can be challenging. Variation in the management of hypertension will be reflected in significant differences hypertension prevalence and control both between and within general practices, regions and nationally. In England the National Institute for Health and Care Excellence (NICE) provides evidence based guidance (EBG) on the management of hypertension [1], reinforced by pay-for-performance (P4P) indicators, the Quality and Outcomes Framework (QOF) [1] [22]. These tools have standardised the recording of BP and provide definitions of what represents uncontrolled BP (Table 1).

**Table 1:** Blood pressure targets in England–(i) NICE National Evidence based Guidance (EBG) and (ii) UK Pay for Performance (PFP) targets.

	Evidence-based guidelines (EBG)	Pay-for-performance (P4P)
**Age < 80 years**	Systolic BP ≤140 mmHg Diastolic BP ≤90 mmHg	Systolic BP ≤150 mmHg Diastolic BP ≤90 mmHg
**Age ≥ 80 years**	Systolic BP ≤140 mmHg Diastolic BP ≤90 mmHg	Systolic BP ≤150 mmHg Diastolic BP ≤90 mmHg
**Type 1 Diabetes Mellitus**	Systolic BP ≤135 mmHg Diastolic BP ≤85 mmHg	Achievement assessed for: Systolic BP ≤150 mmHg Diastolic BP ≤90 mmHg AND Systolic BP ≤140 mmHg Diastolic BP ≤90 mmHg
**Type 1 Diabetes Mellitus with albuminuria or ≥2 metabolic risk factors**	Systolic BP ≤130 mmHg Diastolic BP ≤80 mmHg	
**Type 2 Diabetes Mellitus**	Systolic BP ≤140 mmHg Diastolic BP ≤90 mmHg	
**Type 2 Diabetes Mellitus with retinal, renal or cerebrovascular disease**	Systolic BP ≤130 mmHg Diastolic BP ≤80 mmHg	
**Chronic kidney disease**	Systolic BP ≤140 mmHg Diastolic BP ≤90 mmHg	No disease specific achievement target
**Chronic kidney disease with albumin creatinine ratio ≥70mg/mmol**	Systolic BP ≤130 mmHg Diastolic BP ≤80 mmHg	

We carried out this study to estimate the prevalence of diagnosed hypertension, evaluate its management in relation to attainment of blood pressure control targets, and explore inter-practice variation using the Royal College of General Practitioners (RCGP) Research and Surveillance Centre (RSC) network database. In addition, we identified sociodemographic and practice characteristics associated with. We aimed to demonstrate the extent to which variation in the prevalence of diagnosed hypertension and attainment of blood pressure targets could be explained by these individual and practice level characteristics.

## Methods

### Study design and data source

We carried out a cross-sectional study using data routinely collected from the Royal College of General Practice Research and Surveillance Centre (RCGP RSC) database. English general practice lends itself to this type of research because it is a registration-based system (people register with a single practice) and P4P has standardised the recording of chronic disease data. In addition, practitioners can “exception report” patients from P4P indicators–for example where a patient declines treatment or is taking the maximum tolerated level of medication. This means that patients who decline or can’t tolerate therapy can be differentiated from those in whom treatment could be optimised.

The RCGP RSC is a gold standard surveillance service which collects data from computerised medical records (CMR) systems from more than 200 general practices across England with a registered population of approximately 2 million [23]. The RCGP RSC dataset is a representative network, although there is a small over representation of adults aged 25–44, and underrepresentation of people of white ethnicity, people in IMD quintiles indicative of higher deprivation, and overrepresentation of practices in London [23]. The database includes all recorded clinical codes within the primary care record. These clinical codes include diagnosis codes, medication codes, investigation codes, and process of care codes. Data for this study was extracted from the RCGP RSC database using a predefined list of Read 2 codes[24] as specified below.

### Case definitions

#### Diagnosed hypertension

Individuals with a diagnosis of hypertension were identified by searching the RCGP RSC database for diagnostic and clinical codes for hypertension.

#### Uncontrolled hypertension

The definition of “uncontrolled hypertension” was based on national EBG targets for adults aged ≥ or < 80 years, as per Table 1. The proportion of individuals who had had a blood pressure measurement in the previous 12 months was calculated, as was the proportion who had a blood pressure recorded 12–36 months and >36 months previously. Individuals who had had a blood pressure measurement in the previous 12 months and in whom the latest systolic or diastolic blood pressure was greater than the targets listed above, were categorised as having uncontrolled hypertension. The most recent blood pressure reading was used for each individual. Where more than one blood pressure reading was recording, the lower result was used. This method is consistent with those used to determine attainment of blood pressure control in the P4P indicators [22]. Our principal analysis did not include the disease specific targets (Table 1). However, we conducted a sensitivity analysis removing these patients, as detailed below, to explore if this made a difference.

#### Hypertension treatment

Antihypertensive prescription was determined using drug dictionary codes. The code and date of latest prescription was extracted. Antihypertensive medication was defined as one or more of an Angiotensin converting enzyme inhibitor, an Angiotensin receptor blocker, a calcium channel blocker or a thiazide diuretic (including indapamide).

### Inclusion criteria

All patients aged ≥ 18 years with a diagnosis of hypertension on or preceding 31st December 2016 were included. Individuals with diagnosed hypertension in whom blood pressure had been recorded in the preceding 12 months were included in the primary analysis of blood pressure control.

### Exclusion criteria

Patients no longer registered with a participating practice at the end of the observation period, e.g. those who had moved practice or died, were excluded. Patients with an exception reporting code for hypertension and those who had not had a blood pressure measurement in the preceding 12 months were excluded from the analysis of blood pressure control. This is because UK national guidelines recommend that blood pressure is monitored least annually in people with diagnosed hypertension, and we aimed to demonstrate variation in attainment of blood pressure targets in people with diagnosed hypertension who were being actively managed in primary care. Any patients who have codes suggesting they declined any form of data sharing are not analysed by RCGP RSC (approximately 2.2%).

### Sensitivity analysis

Owing to the complexity of the differential EBG blood pressure targets recommended in individuals with co-morbidities, e.g. chronic kidney disease, diabetes mellitus, we did not consider these within the main analysis of uncontrolled hypertension, which is likely to somewhat underestimate the prevalence of uncontrolled hypertension. It could also be expected that individuals with co-morbidities would have more proactive blood pressure management, owing to their higher risk of complications, and therefore inclusion of these individuals could underestimate variation. Therefore, to validate our approach we repeated the analysis excluding those people in whom lower blood pressure targets are recommended by EBG and confirmed the position of practices outside the funnel plot control limits relative to the initial model. These individuals were identified using clinical codes for diabetes mellitus, chronic kidney disease, cardiovascular disease etc.

As described above the main analysis included only those with a blood pressure recorded within the previous 12 months. A further sensitivity analysis was undertaken to test the effect of including individuals in who were excluded from the main analysis due to missing blood pressure data–i.e. those in whom the latest blood pressure reading was >12 months previously.

### Statistical analyses

Descriptive statistics were used to describe the proportion of people with hypertension and uncontrolled hypertension. Mean and standard deviation were used to describe continuous variables and proportions with confidence intervals for categorical variables.

Logistic regression was used to calculate the indirectly standardised prevalence of hypertension, and of uncontrolled hypertension at GP practice level. This is an established method of calculating indirectly standardised ratios and allows the input of explanatory variables beyond age and gender [25]. Models were adjusted for age, sex, ethnicity, index of multiple deprivation (IMD), body mass index (BMI), co-morbidities, prescription of anti-hypertensive medication, NHS region and practice size. Funnel plots were used to demonstrate variation in the proportion of patients with detected and uncontrolled hypertension at a practice level, again a widely used method of graphically representing performance and can be used to compare institutions [26]. The 95% and 99.8% control limits, or ‘funnels’, represent natural variation around the average value. Observation points which fall within the control limits have a confidence interval which includes the average value and therefore this variation is expected. In contrast those which fall outside the control limits do not contain the average value within their control limits, and therefore may indicate unexpected variation [27]. In a well-adjusted model we expect 95% and 99.8% of observations will fall within the respective control limits. Over-dispersion was defined as present where the number of observations outside the control limits is substantially higher than this [28]. In our study we have indirectly standardised against the RCGP RSC population. Therefore comparisons between individual practices and the whole population are possible, whereas comparisons between practices are not. The model C statistic was calculated, to provide a measure of model fit.

Logistic regression analysis was used to identify sociodemographic and practice characteristics associated with uncontrolled hypertension. Odds ratios are reported adjusted for age, gender, IMD quintile, ethnicity, multimorbidity, practice size and NHS region of the practice.

Statistical analysis was carried out R studio version 3.2.5 (2016-04-14).

### Ethics

The data used for the analysis was pseudonymised at the point of extraction, and encrypted prior to uploading to the secure network on which this analysis took place. Potentially personal data were not identifiable during the analysis. This study was approved by RCGP and was classified as an audit of current practice using the Medical Research Council and Health Research Authority research categorisation tool [29]. Therefore, no further ethical approval is required.

## Results

**Fig 1:**
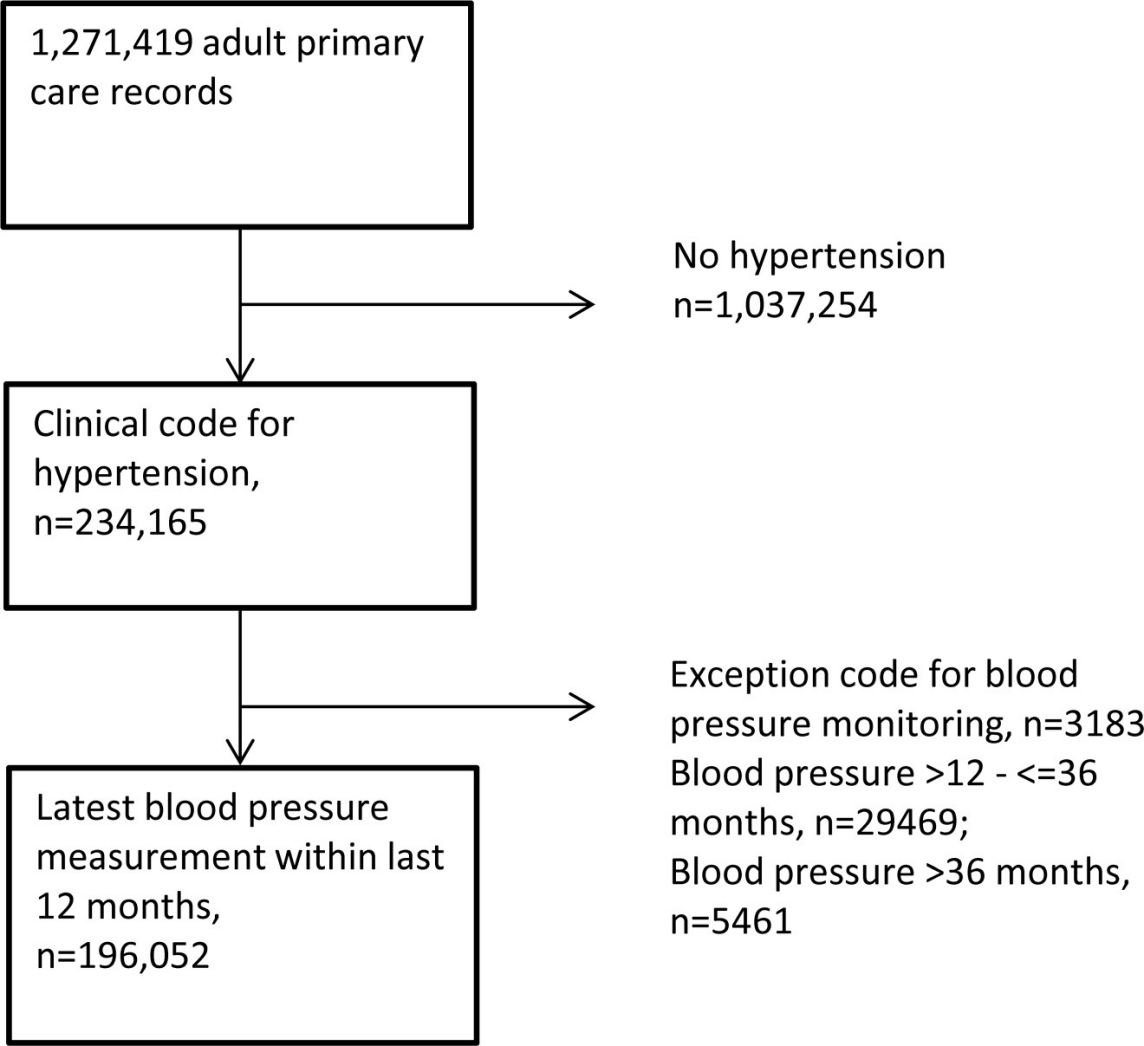
Study inclusions and exclusions.

Data was extracted from the primary care records of 1,271,419 adult men and women registered at 164 general practices in England. Study inclusions and exclusions are shown in Fig 1.

The crude prevalence of diagnosed hypertension within the RCGP RSC cohort was 18.4% (18.4–18.5), n = 234,165. The latest blood pressure reading was taken within the last 12 months in 196,052 individuals (84.9%). The latest blood pressure reading was obtained between 12 and 36 months in 29469 individuals (12.8%), and >36 months previously in 5461 individuals (2.4%).

The latest blood pressure reading demonstrated controlled blood pressure in 146,553/196,052 individuals, 74.8% (74.6–74.9) while 49499, 25.2% (25.1–25.4) had uncontrolled hypertension.

### Practice prevalence of diagnosed hypertension

**Fig 2:**
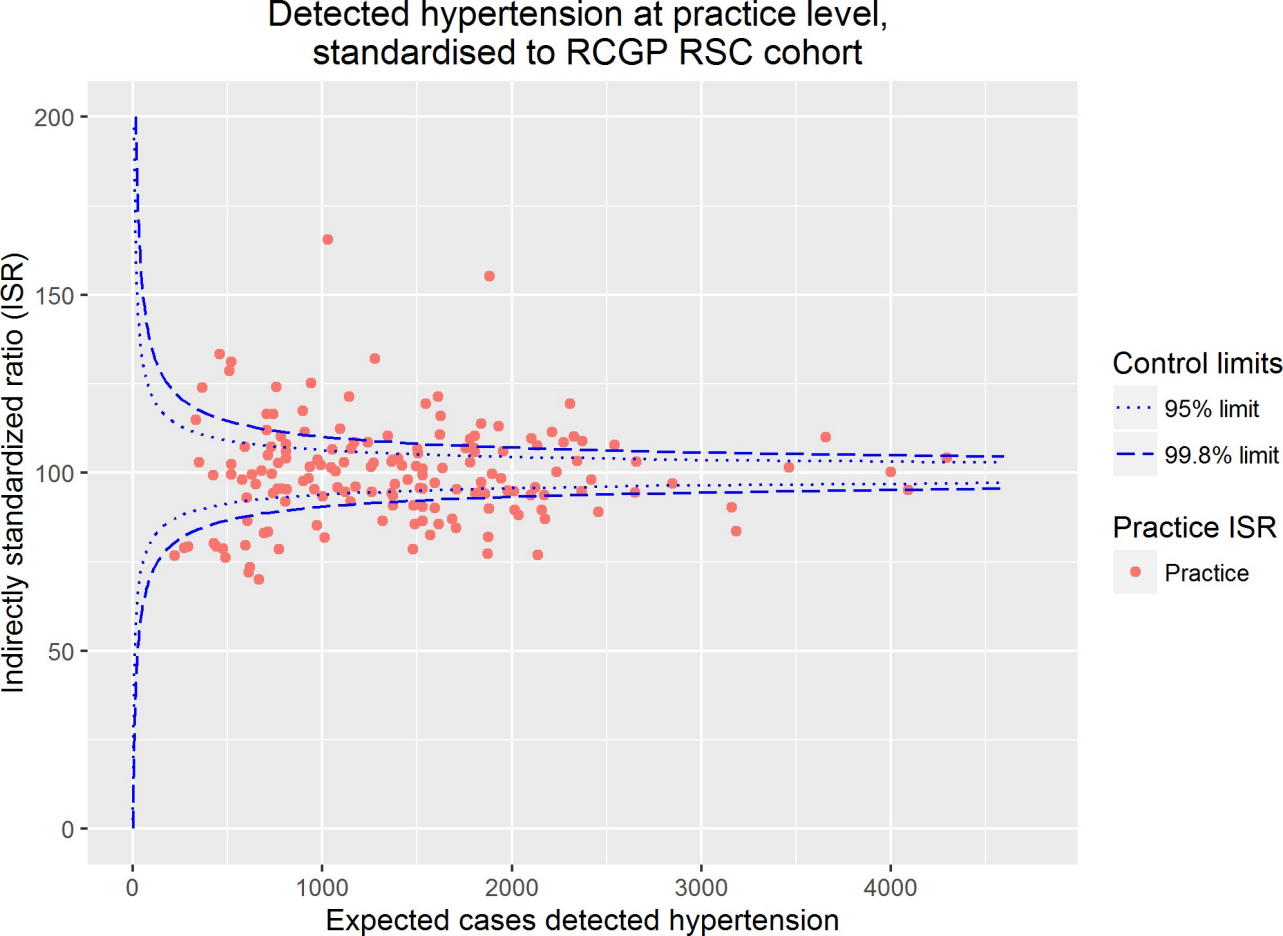
Prevalence of detected hypertension in primary care at practice level, standardised to the RCGP RSC cohort.

The crude practice prevalence of diagnosed hypertension in adults ranged from 13.5% to 38.4%. The indirectly standardised ratio (ISR) for detected hypertension was calculated, adjusted for age, sex, ethnicity, deprivation, practice size and practice location, and ranged from 70.0–165.5 (C statistic 0.88). Of the 164 practices within the sample 40.2% (n = 66) fell within the 95% control limits of the funnel plot, and 54.2% (n = 89), fell within the 99.8% control, as shown in Fig 2. Therefore, in approximately 60% of practices the prevalence of diagnosed hypertension fell beyond the 95% control limts and in approximately 45% this prevalence fell beyond the 98% limits, demonstrating substantial over-dispersion despite adjustment for a range of individual and practice characteristics.

### Practice prevalence of uncontrolled hypertension

**Fig 3:**
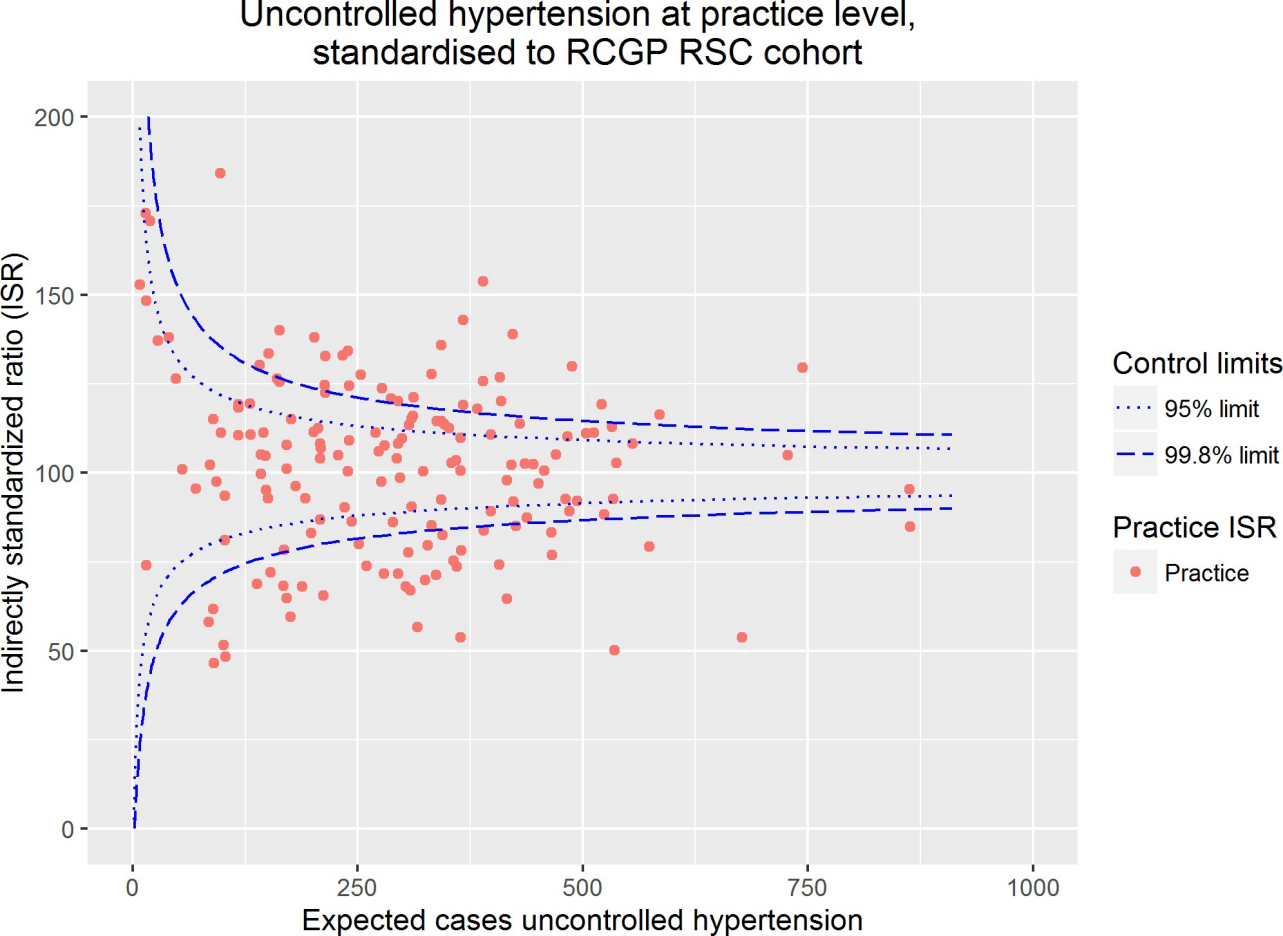
Prevalence of uncontrolled hypertension in primary care at practice level, standardised to the RCGP RSC cohort.

The crude practice prevalence of uncontrolled hypertension in adults ranged from 11.8–47.9%. The ISR for uncontrolled hypertension was calculated, adjusted for age, sex, ethnicity, deprivation, co-morbidity, obesity, anti-hypertensive medication prescription, practice size and practice location, and ranged from 46.5.0–184.2 (C statistic 0.622). The proportion of practices within the 95% control limits was 40.2% (n = 66) and the number within the 99.8% control limits was 59.1% (n = 97)), as shown in Fig 3. As with the prevalence of diagnosed hypertension, the funnel plots demonstrate substantial overdispersion despite adjustment for individual and practice characteristics.

### Sensitivity analysis

**Fig 4:**
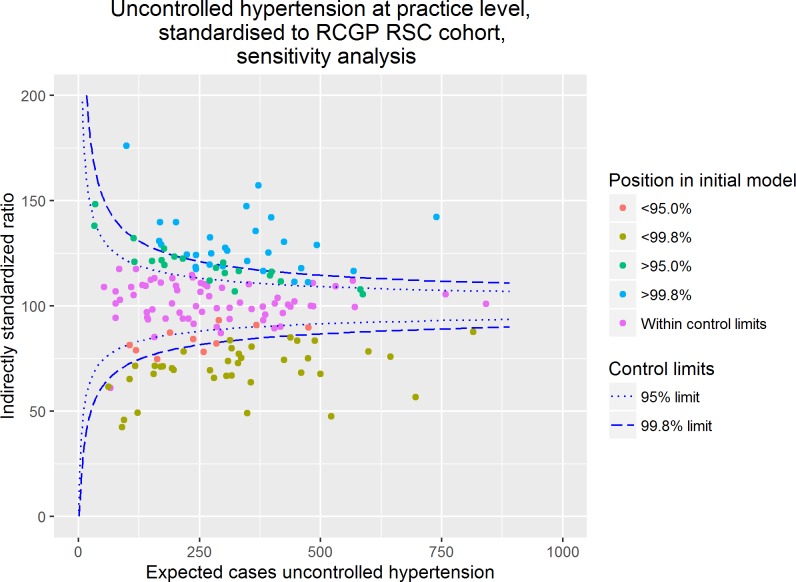
Sensitivity analysis—Uncontrolled hypertension in primary care at practice level excluding patients in whom tighter blood pressure targets are recommended, standardised to the RCGP RSC cohort.

Following exclusion of patients in whom tighter blood pressure control is recommended by EBG the crude practice prevalence of uncontrolled hypertension in adults ranged from 11.8–50.4%. Consistent with the results demonstrated in Fig 3, there was substantial overdispersion. The proportion of practices falling within the 95%, n = 66 practices, and 99.8%, n = 99, control limits was similar to the intial model. As demonstrated in Fig 4, in the sensitivity analysis most of practices remained in the same position relative to the funnel plot control limits.

**Fig 5:**
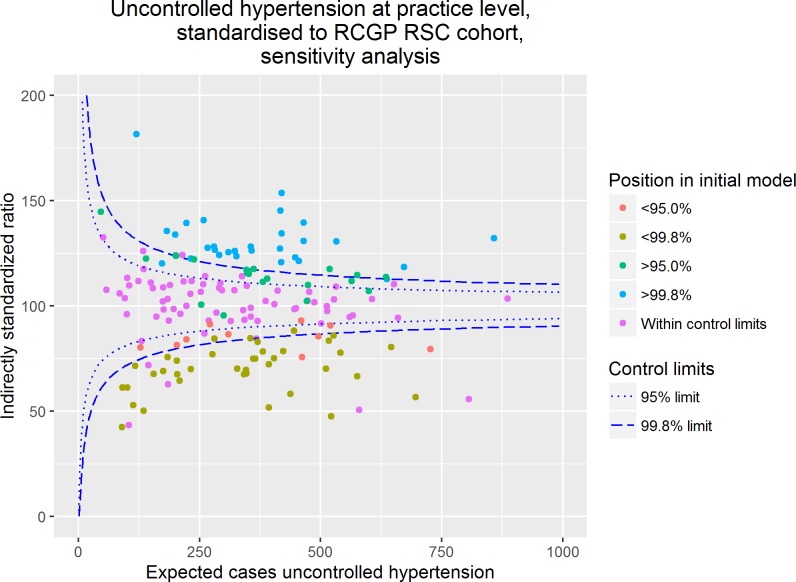
Sensitivity analysis—Uncontrolled hypertension in primary care at practice level in adults with diagnosed hypertension and a blood pressure reading at any time post diagnosis, standardised to the RCGP RSC cohort.

The results of an additional sensitivity analysis to test the impact of excluding all individuals in whom the latest blood pressure recording was >12 months previously. The ISR for uncontrolled hypertension was calcuated using the same adjustment as per Fig 3. Fig 5 demonstrates the practice ISR for uncontrolled hypertension when all individuals with a blood pressure measurement after diagnosis are included. Inclusion of all individuals did not materially change the results in regards to dispersion. The proportion of practices falling within the 95%, n = 59 practices, and 99.8%, n = 99, control limits was similar to the intial model.

### Factors associated with uncontrolled hypertension–multivariable analysis

#### Individual characteristics associated with uncontrolled hypertension

Age, gender and ethnicity were all significantly associated with having uncontrolled hypertension (see Table 2). By comparison to individuals aged 18–35 uncontrolled hypertension was significantly more frequent in adults aged 36–80 years old. In contrast adults aged over 80 years were significantly less likely to have uncontrolled hypertension, adjusted OR 0.39 (0.36–0.43). Female gender was associated with a reduced likelihood of having uncontrolled hypertension, 0.95 (0.93–0.97). By comparison to those of white ethnicity, uncontrolled hypertension was less common in people of Asian ethnicity, and was more common in people of black and unknown ethnicity.

**Table 2:** Individual and practice level characteristics associated uncontrolled hypertension adjusted for age, sex, ethnicity, deprivation, co-morbidity, obesity, hypertension prescription, practice size and practice location.

	Odds Ratio	95% confidence interval		P value
Age (years)				
18–35				
36–50	1.09	(0.99,	1.21)	0.08
51–65	0.98	(0.89,	1.08)	0.76
66–80	0.94	(0.85,	1.03)	0.21
>80	0.39	(0.36,	0.43)	<0.001
Sex				
Male				
Female	0.95	(0.93,	0.97)	<0.001
Ethnicity				
White ethnicity				
Asian ethnicity	0.86	(0.81,	0.91)	<0.001
Black ethnicity	1.19	(1.12,	1.27)	<0.001
Mixed ethnicity	1.12	(0.99,	1.28)	0.08
Other ethnicity	0.97	(0.83,	1.14)	0.72
Unknown ethnicity	1.13	(1.10,	1.16)	<0.001
Index of multiple deprivation (IMD)				
IMD Quintile 1 (most deprived)				
IMD Quintile 2	1.05	(1.01,	1.09)	0.02
IMD Quintile 3	1.08	(1.04,	1.12)	<0.001
IMD Quintile 4	1.05	(1.01,	1.09)	0.01
IMD Quintile 5 (least deprived)	1.01	(0.98,	1.05)	0.47
**BMI Category (kg/m**^**2**^**)**				
BMI Normal (18.5-<25.0)				
BMI Underweight (<18.5)	1.04	(0.93,	1.17)	0.50
BMI Overweight (25.1-<30.0)	1.09	(1.06,	1.13)	<0.001
BMI Obese (30.1-<40.0)	1.21	(1.18,	1.25)	<0.001
BMI Severely Obese (>40.0)	1.42	(1.35,	1.49)	<0.001
**Co-morbidities**				
No physical co-morbidities				
One physical comorbidity	0.80	(0.77,	0.82)	<0.001
Two or more physical co-morbidities	0.59	(0.58,	0.61)	<0.001
**No. prescribed anti-hypertensives**				
None				
1–2 anti-hypertensives prescribed	0.99	(0.96,	1.02)	0.52
3+ anti-hypertensives prescribed	1.25	(1.20,	1.31)	<0.001
**NHS Region**				
NHS London				
NHS North	1.13	(1.08,	1.17)	<0.001
NHS Midlands and East	0.97	(0.94,	1.01)	0.17
NHS South	1.12	(1.08,	1.17)	<0.001
**Practice Size**				
No. Registered patients (continuous)	<1.00	(1.00,	1.00)	<0.001

Deprivation, measured by IMD quintile, a mixed pattern was seen. Individuals in IMD quintiles 2, 3 and 4 were more likely than those in IMD quintile 1 (most deprived) to have uncontrolled hypertension. In contrast individuals in IMD quintile 5 (least deprived) were no more or less likely to have uncontrolled hypertension than those in in quintile 1. People who were overweight or obese were more likely than those with a normal BMI to have uncontrolled hypertension.

When age and co-morbidity were considered together, the presence of one physical co-morbidity was not associated with uncontrolled hypertension in any age band. However, individuals aged > 35 with two or more comorbidities were less likely than those aged 18–35 years and without comorbidity to have uncontrolled hypertension. This is in contrast to the relationship between age and uncontrolled hypertension when age was considered as a distinct explanatory variable.

Prescription of anti-hypertensives was associated with blood pressure control. People prescribed 1–2 anti-hypertensives were less likely than those on no medication to have uncontrolled hypertension, and those prescribed three or more anti-hypertensives were more likely to have uncontrolled hypertension compared to people taking no medication.

#### Practice characteristics

Uncontrolled hypertension was associated with smaller sized practice, although the effect size was small. Individuals registered at practices in NHS regions South and Midlands and East were more likely to have uncontrolled hypertension than those in NHS London.

Factors associated with uncontrolled hypertension (Table 2).

## Discussion

In this cross-sectional of study of 1,271,419 adults we have demonstrated considerable inter-practice variation in the prevalence of diagnosed hypertension and its control. At practice level there was a three-fold difference between the practice with the lowest and the highest prevalence of hypertension and a four-fold difference between the lowest and highest prevalence of uncontrolled hypertension. This variation is only partially explained by routinely collected sociodemographic and practice level data. This makes it challenging to differentiate between potentially modifiable from unmodifiable causes of variation and to identify the root cause of variation.

The greatest gap in effective management of hypertension was in men and people of black ethnicity have worse control, while people of Asian ethnicity had better blood pressure control better. These observations point towards the need to improve hypertension management and make it more consistent between practices. The differences between gender and ethnicity may provide some insight into where further exploratory studies should be conducted.

### Comparison with the other studies

#### Variation in hypertension diagnosis and its control

Unwarranted variation can contribute to inefficiency through over- and under-utilisation of healthcare, however distinguishing warranted from unwarranted variation is challenging [17] [30]. Factors can lead to variation including individual factors such as clinician decision making [31] [32] [33], local cultural beliefs of clinicians and patients [33], and system factors such as incentives and system delivery factors [33] [34]. In a systematic review of variation research in OECD countries only 10% of studies focused on the underlying cause of variation [34].

Our data show marked variation in the prevalence of diagnosed hypertension and its control which is not fully explained by controlling for routinely collected explanatory variables. This is consistent with previous UK studies which have demonstrated marked variation in practice achievement of hypertension targets [35]. In our study, despite adjustment, only 66 (40.2%) of 164 practices lying with the 95.0% control limits for hypertension prevalence and 97 (59.1%) of practices lying within the 99.8% control limits for uncontrolled hypertension (Figs 2 and 3). This over-dispersion usually indicates that the variation demonstrated is not fully explained by the explanatory variables included within a model [28]. In this study we have been able to adjust for practice size and location. A North-South divide in terms of mortality has been demonstrated in the UK, with individuals living in Northern regions experiencing higher mortality than Southern counterparts [36]. Similarly, practice size is associated with quality in the UK, and smaller practices have been shown to score less on quality indicators than larger practices [37]. However, despite the adjustments in our models variation is not fully explained. This may reflect variation in processes of care or practice characteristics, which are not routinely collected. Determining the importance of such factors and their practical impact on care quality is difficult [28]. While measuring patient sociodemographic factors is easy, measuring their preferences for or against a particular treatment option is not [38]. Moreover, system level factors (e.g. recall register) and structural factors (e.g. clinician to patient ratios) are not routinely available and therefore cannot be considered when evaluating the causes of variation. Our results highlight that there are limitations in attempting to quantify variation without access to sufficient individual, structural and context factors. This is important because efforts to reduce variation may be ineffective, or worse counter-productive, where the proportion of variation which is genuinely unwarranted and the cause of the unwarranted variation is unknown [17].

Our results demonstrate several factors associated with poor blood pressure control. Uncontrolled hypertension was more frequent among men compared to women, people of black and of Asian ethnicity compared to people of white ethnicity, people who were overweight and obese compared to people of normal weight and people taking three or more anti-hypertensive medications compared to those on no medications. Regarding practice characteristics, uncontrolled hypertension was found more frequently in patients registered at practices in NHS Midlands and East and in NHS South, compared to London. Uncontrolled hypertension was significantly more frequent in larger practices, although the difference was small.

Uncontrolled hypertension was less frequent in people aged over 80 years compared to those aged 18–35 years, adjusted Odds Ratio (95% Confidence interval) 0.39 (0.36–0.43), and in people with co-morbidities compared to those without physical co-morbidities. An association [35] between co-morbidity and achievement of hypertension P4P targets has been demonstrated in a previous English cross-sectional study. There are a number of possible explanations for this finding. There is significant overlap in the pharmacological agents used to treat cardiac disease, chronic kidney disease and hypertension [1] [39] [40] [41] [42]. Therefore it would be reasonable to expect a person with co-morbid cardiac or renal disease to be more likely to be prescribed agents which would directly or indirectly treat their hypertension than someone without these conditions. Additionally, it is well established that future risk of cardiovascular disease is influenced by the presence or absence of risk factors including as chronic disease (e.g. chronic kidney disease, diabetes, hypertension[43] [44]. The importance of managing blood pressure is emphasised in the presence of additional cardiovascular risk factors [39]. Therefore, our findings may reflect more intensive blood pressure management in individuals with higher cardiovascular risk. Another explanation for our findings could relate to frequency of attendance in primary or secondary care services. There is evidence from the UK that people living with co-morbidities account for a disproportionate number of primary care attendances, outpatient appointments and emergency admissions [45] [46]. Additionally, regular clinical reviews, including blood pressure assessment, are recommended by NICE in the management of several chronic conditions including Type 1[40] and Type 2 Diabetes[41], COPD[47] and chronic heart failure[48]. It is possible that the increased clinical input received by people attending primary and/or secondary care more regularly is associated with more opportunities to review and adjust blood pressure management as appropriate, although this has not been demonstrated previously.

### Strengths and weaknesses of this study

A key strength of this study is its use of computerised medical records from a nationally representative sample of general practices, with over 1.2 million adults. Accordingly these results are generalizable to the wider English population. An important weakness of this study is that in comparing the practice prevalence of controlled and uncontrolled hypertension we are only able to control for explanatory variables which are routinely collected. We are therefore unable to control for structural and process factors which may be important, for example full time equivalent practice clinical staff per patient population, or the use of allied health professionals in blood pressure management. We are, however, highlighting this particular weakness as something which is frequent in attempts to identify unwarranted variation[34] and call for more research to identify the role of these complex factors in mediating variation. Another important limitation is that the case definition for ‘uncontrolled hypertension’ relies on blood pressure measurements within the primary care setting. White-coat hypertension may contribute to transient high blood pressure readings when these are obtained with a general practice. UK national guidance recommends ambulatory or home blood pressure monitoring for individuals with white-coat hypertension[1] and practice would be expected to input these results into the clinical record. However, remote blood pressure monitoring may not always be available therefore the prevalence of uncontrolled hypertension in our study may be somewhat overestimated. However, the methods we have used are consistent with UK pay-for-performance data extraction logic.

### Interpretation of findings and implications for clinicians and policy makers

The practice level variation which we have demonstrated is not fully explained by individual and practice level characteristics captured in our routine datasets. This suggests that additional contextual factors may account for variation. This is important in which reducing unwarranted variation has become synonymous with quality improvement in the dialogue of national and local healthcare commissioners [17] [30]. Efforts to reduce variation may be ineffective, or worse counter-productive, where the proportion and the cause of the unwarranted variation is unknown [17]. While routine datasets provide rich information their limitations, in particular in regards to measuring processes of care delivery and context, must be recognised, particularly when they are being used to inform local healthcare priorities. It is vital that efforts to reduce unwarranted variation recognise that we currently lack an understanding of the underlying causes. This is likely to require measurement of process and structural factors not currently included in routine datasets.

## Conclusions

Reducing unwarranted variation in hypertension management is likely to improve the diagnosis of hypertension and its control and contribute to better health outcomes. However, routine data sources alone are not sufficient to distinguish warranted from unwarranted variation and do not capture sufficient contextual factors to identify the causes of variation. Organisations and researchers seeking to understand variation should consider mixed methods to explore the wider contextual and process factors which may contribute to variation.

## Supporting information

S1 File Strobe checklist. (DOC)
